# Assessment of Anterior Cruciate Ligament Tibial Footprint Sagittal Diameter and Orientation of the Ligament in the Intercondylar Notch in Indian Population: A Magnetic Resonance Imaging (MRI) Analysis

**DOI:** 10.7759/cureus.7511

**Published:** 2020-04-02

**Authors:** Balgovind S Raja, Varun Garg, Souvik Paul, Sukhmin Singh, Watson Thomas, Siddharth R K, Aman Verma

**Affiliations:** 1 Orthopaedics, All India Institute of Medical Sciences Rishikesh, Rishikesh, IND

**Keywords:** anterior cruciate ligament, sagittal centre, tibial foot print, roof angle, acl inclination angle, acl-bluemensaat angle

## Abstract

Aim: To study the relevant anatomy of anterior cruciate ligament tibial footprint and orientation of the ligament in the intercondylar roof in Indian population the using MRI.

Methods: A total of 70 knee MRI with intact anterior cruciate ligament (ACL) was assessed for intercondylar roof angle, ACL inclination angle, ACL-bluemensaat angle, ACL sagittal center, and tibial insertion size.

Results: The ACL tibial sagittal center was found to be at 43.5% of the anteroposterior tibial length. Tibial insertion size averaged 15.40 (±1.29) mm with no significant difference in males and females (p > 0.05). The roof angle was 36.29 (± 4.02) ˚ and the ACL inclination angle and ACL-bluemensaat angle were 51.22 (± 3.39) ˚ and 4.70 (±3.35) ˚ respectively with no significant sex difference (p > 0.05).

Conclusion: The ACL tibial insertion size averaged 15.40 mm and its center was at 43.51% along the Staubli and Rauschning line. The mean roof angle was 36.29 degrees and the ACL-bluemensaat angle was 4.70 degrees. Understanding of the tibial footprint morphology and the relation of the ligament to the roof of the intercondylar notch helps in anatomical graft placement during reconstruction.

## Introduction

Incidences of ligament injuries in the population are on the rise due to increased involvement in sports activities [1]. Anterior cruciate ligament (ACL) tears are among the most common ligament injuries in the knee joint and usually are seen secondary to excessive valgus stress, forced external rotation of the femur on a fixed tibia, and hyperextension [2]. Reconstruction of ACL owing to innovations in surgical instruments and improved surgical techniques is widely performed and understanding the anatomy of the ACL tibial footprint, the orientation of the ligament in relation to the roof of the intercondylar fossa and the tibial surface helps in precise anatomical placement of the graft in ACL reconstruction [3-5].

 Ethnic variations in Indian knees have been thoroughly documented. Vaidya et al. observed the variations in dimensions of the bone geometry in the Indian scenario and Mullaji et al. documented the rotational axes differences of the distal femur in relation to the Caucasian population [6-7]. Though the anatomy of the ACL is well defined in literature, currently in the Indian scenario there appears to be little literature regarding the orientation of native ACL in the intercondylar notch and its tibial foot anatomy. A comprehensive idea regarding the tibial insertion anatomy, its variations along with relations of ACL to the intercondylar roof, and its inclination to the tibial surface will guide the surgeon to achieve an accurate and impingement-free graft placement.

The purpose of our study was to analyze the native ACL relations to roof of the intercondylar fossa, tibial surface and to assess the distal tibial insertion site morphology using MRI in the Indian population.

## Materials and methods

The study included 70 skeletally mature subjects (40 males and 30 females) selected retrospectively with normal knee MRI after the approval from the ethics committee (IEC2/OUT/44/18). Exclusion criteria included knees with osteoarthritic changes (Kellgren-Lawrence grade ≥ 2), presence of epiphyseal line, concomitant ligament injuries, abnormalities of extensor mechanism, history of previous surgery or fractures around knee, and history of patellar dislocation or subluxation. MRI was performed employing 1.5 T scanner with proton density fat suppression, coronal plane, 4 mm slice thickness, 0.5 mm space, matrix of 352 x 224, 13.6 ms echo time, and repetition time of 1700-2000 ms. Sagittal and axial images, thickness of 3.5 mm, space of 0.5 mm, matrix of 352 x 224, echo time of 86 ms, repetition time of 4200 ms with the knees in near normal extension.

**Figure 1:**
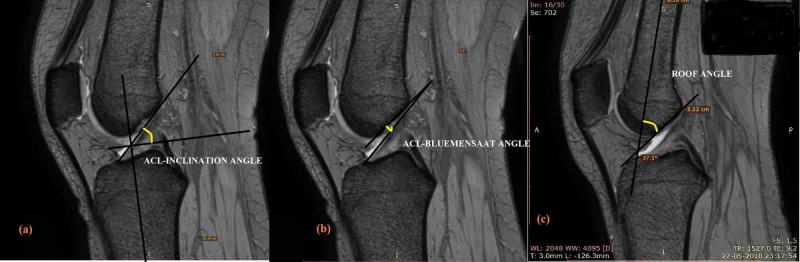
Illustrations showing measurement of ACL-inclination angle, ACL-bluemensaat angle, and roof angle. T2 sagittal image slices showing the ACL (anterior cruciate ligament) relationship. (a) Angle between the tangent to the anterior aspect of the ACL and the perpendicular to the tibial axis is the ACL-inclination angle; (b) ACL-bluemensaat angle is formed by the blumensaat line and the tangent to the anterior aspect of ACL; (c) angle measure between the femoral axis and bluemensaat line gives roof angle.

Sagittal T2W slice in which the ACL tibial insertion fibers are well seen was selected for determining the relations of ACL. Femoral and tibial axes were drawn first joining the midpoints of two lines in their respective diaphysis. The angle made by the bluemensaat line and the femoral axis is defined as the roof angle [8] (Figure 1) and the ACL-bluemensaat angle refers to the angle formed by the bluemensaat line and the tangent to the anterior aspect of the anterior cruciate ligament (Figure 1) [9]. The ACL inclination angle is formed by the tangent to the anterior aspect of the ACL fibers and the perpendicular to the tibial axis at the ACL insertion site [10] (Figure 1). Femoral and tibial axes are drawn joining the midpoints of two lines in their respective diaphysis.

**Figure 2:**
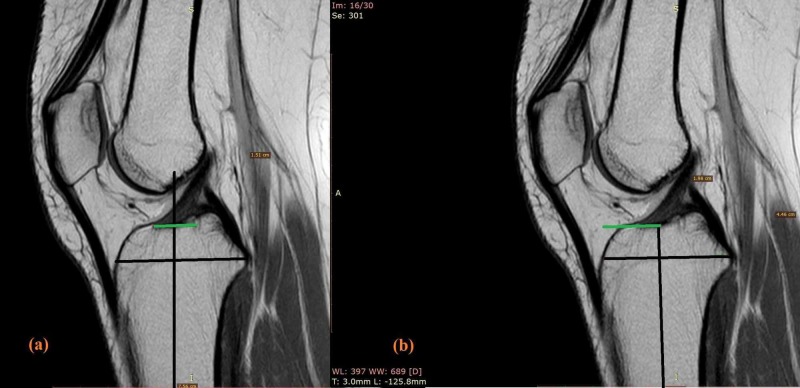
T1 sagittal slices showing ACL tibial footprint anatomy. The tibial foot print sagittal length is measured along the Staubli and Rauschning line (a) and the ACL (anterior cruciate ligament) sagittal center is measured from the anterior aspect to the center of ACL tibial footprint along the same line (b) and is expressed as percentage.

Both T1- and T2- weighted sequences on the sagittal projection were evaluated to discover the ACL tibial insertion size and sagittal ACL tibial center. The sagittal slice that contained the largest tibial footprint was selected. The length along the Staubli and Rauschning line refers to the insertion site length (Figure 2). The percentage of the anteroposterior length from the anterior tibial cortex to the center of the ACL along the Staubli and Rauschningline gives sagittal ACL center (Figure 2) [10].

Measurements were made using Radiant Dicom software version 4.6.9, copyright 2009-2018 Medixant. The data obtained were assessed using Microscoft Excel software and XL STAT software. Shapiro-Wilk test was used to assess the normality of the sample and Student’s *t* test was employed to compare the difference between genders. The values were expressed as mean +/- standard deviation and a p value of <0.05 was considered statistically significant.

## Results

The average age of the subjects was 35 with 18-51 years range. The roof angle was 36.29 (±4.02) ˚ with no significant difference in males and females (p > 0.05), (Table 1). The ACL inclination angle averaged 51.22 (±3.39) ˚ with 51.72 (±3.16) ˚ in males and 50.53 (±3.66) ˚ in females respectively (p > 0.05). The mean ACL-bluemensaat angle was found out to be 4.70 (±3.35) ˚ with no significant difference between the sexes (p > 0.05). Tibial insertion site was found out to be 15.40+/-1.29 along the Staubli and Rauschning line with 15.63+/-1.47 in males and 15.08+/-0.92 in females respectively (p > 0.05). The ACL sagittal center averaged 43.51%+/-3.1 from the anterior cortex along the same reference line with 43.72%+/-3.3 in males and 43.22%+/-2.9 in females (Table 1), (p > 0.05).

**Table 1:** Patient demographics and study results. *Length measured along the anteroposterior diameter of proximal tibia in millimeters, **p value < 0.05 is significant. ACL, anterior cruciate ligament.

Parameters	Male	Female	Total	p value**
Number of subjects	40	30	70	-
Roof angle˚				
Mean	36.96	35.36	36.29	0.059
Standard deviation	3.90	4.09	4.02	
ACL-inclination angle˚				
Mean	51.72	50.53	51.22	0.240
Standard deviation	3.16	3.66	3.39	
ACL-bluemensaat angle˚				
Mean	4.66	4.75	4.70	0.384
Standard deviation	3.47	3.25	3.35	
Tibial insertion site*				
Mean	15.63	15.08	15.40	0.067
Standard deviation	1.47	0.92	1.29	
ACL tibial footprint sagittal center*				
Mean	43.72%	43.22%	43.51%	0.781
Standard deviation	3.3	2.9	3.1	

## Discussion

The principal findings of our study include: a) The ACL tibial footprint sagittal center averaged 43.51% along the AP width of tibia; b) The average ACL tibial footprint sagittal insertion length was 15.4 mm; c) The intercondylar roof angle averaged 36.29˚; d) The ACL-inclination angle and ACL-bluemensaat angle averaged 51.22˚ and 4.70˚ respectively.

The proper placement of the tibial tunnel during ACL reconstruction is essential as it would minimize complications like graft impingement, instability, loss of knee extension, and anterior knee pain [11-12]. Howell and Major in their study advocated the ideal tibial tunnel placement to be 44% of the anteroposterior diameter of joint line [11]. Iriuchishima et al. in his study reckoned that if the graft is being placed in anatomic position, with single or double bundle reconstruction techniques the resultant knees experienced an impingement-free environment and more biomechanical stability [13]. A posteriorly placed tunnel would result in a vertically oriented graft which may not provide rotational stability [12]. An anteriorly placed tibial tunnel would lead to a more horizontal graft and in turn lead to impingement and graft failures. The understanding of the tibial insertion site becomes even more necessary in revision cases where the footprint anatomy may be altered. There is currently no literature on ACL tibial footprint morphology in Indian population. Our study revealed the tibial footprint to be about 43.51% ± 3.1 from the anterior tibial cortex along the Staubli and Rauschning line. It is in accordance with the previous studies (Table 2) and was noted to be more than 40% as per Cho et al. study in the East Asian population [14]. It would be advisable to keep the center of the tibial tunnel during single bundle reconstruction between the range 37.31% and 49.71% (mean ± 2SD) as it would help in preventing a very anterior or posteriorly placed graft. This will be extremely practical in revision cases and one needs to be careful in revision of cases wherein the tunnels that are outside this range.

**Table 2:** Summary of literature on ACL tibial footprint. *Value in mm. **Value <0.05 is significant. ACL, anterior cruciate ligament; MRA, magnetic resonance arthrography.

	Our study	Ichiba et al. [21]	Kim et al.	Frank et al. [22]	Scheffel et al.	Staubli et al.
Place of study	India	Japan	South Korea	USA	USA	
Method	MRI	MRI	MRI	MRI	MRI	MRA
Sample size	70	100	164	100	138	35
ACL sagittal insertion size mean*	15.40	15.2	12.4	-	-	-
Standard deviation	1.39	1.9	Not mentioned	-	-	-
p value**		0.453	-	-	-	-
ACL sagittal center mean (%)	43.51	-	-	46	44.1	44.1-males 43.7- females
Standard deviation	3.1	-	-	4	3.4	Not mentioned
p value**		-	-	<0.001	0.228	-

Tibial insertion sites of sagittal diameter < 14 mm favor single bundle reconstruction over double bundle reconstruction, whereas the double bundle reconstruction is typically done when the insertion sites are > 14 mm [15]. Our study revealed the tibial insertion site to be 15.40+/-1.29 mm with no statistical difference between the genders (Table 1). Staubli et al. recorded similar mean tibial insertion size to ours [10].To our best knowledge this is the first study that assesses the tibial footprint anatomy in Indian population.

Illingworth et al. quoted the use of the femoral tunnel angle measured in weight-bearing X-rays and patient’s native ACL inclination angles to assess the anatomic placement of tunnels in ACL reconstruction [16]. Gentili et al. noticed a mean angle of 55.6˚ and Kim et al. found out 55.5+/-6.7˚ in females and 53.9+/-5.8˚ in males for native ACL [9, 17]. Ayerza et al. suggested the sagittal graft inclination angles to be essentially higher than intact ACL [18]. An increased inclination angle paralleled with nonanatomically placed grafts. A value of 55˚ or more was considered as a reliable predictor for nonanatomic placement of femoral tunnel [16]. The inclination angle found in our study population was 51.22+/-3.39˚ (Table 1).

For the graft to enjoy an impingement-free environment, it should be placed in an anatomic position wherein it will be almost parallel to the intercondylar roof. The impingement of the ACL against the roof constitutes the primary mechanism of tears [4]. An increase in roof angle makes the ACL relatively more horizontal which may lead to frequent ruptures in extension due to impingement against the anterior part of the intercondylar notch. A decreased roof angle has ACL in a more vertical position wherein, narrow intercondylar notch may represent the cause of impingement [19].

The ACL-bluemensaat angle relates to the relationship between the roof and the graft. A decreased angle may suggest an anteriorly placed tibial tunnel where the graft is almost parallel to the bluemensaat line or the roof of the intercondylar notch. The angle is considered as an indirect sign for ACL tear in MRI. Gentili et al. recorded the angle to be 1.6˚ whereas, Saxena et al. found it to be 7.06˚ [9, 20]. The only notable study that relates the antecedent parameter in Indian population is by Saxena et al. [20]. The value of their study is questionable when one considers the number of subjects (normal) n = 23 they included in the normal group. Our study revealed the mean angle to be 4.70+/-3.35˚ with no significant difference between males and females (Table 1). Summary of studies concerning the present study is produced in Tables 2 and 3.

**Table 3:** Summary of literature related to ACL relation within knee. *Value < 0.05 is significant. ACL, anterior cruciate ligament.

	Our study	Scheffel et al.	Saxena et al.	Gentili et al.	Ayerza et al.
Place of study	India	USA	India	USA	Argentina
Method	MRI	MRI	MRI	MRI	MRI
Sample size	70	138	24	35	30
Roof angle mean˚	36.29	34.7	44.24	-	-
Standard deviation	4.02	5.2	3.64	-	-
p value*		0.026	<0.001	-	-
ACL-inclination angle mean ˚	51.22	-	51.34	55.6	51
Standard deviation	3.39	-	3.99	Not mentioned	Not mentioned
p value*		-	0.886	-	-
ACL-bluemensaat angle mean ˚	4.70	-	7.06	1.6	-
Standard deviation	3.35	-	1.44	Not mentioned	-
p value*		-	0.0012	-	-

The study has its own limitations. First, patient height and weight were not recorded as the study was retrospective in nature. Second, the femoral insertion site morphology and ACL medio-lateral width being difficult to appreciate in MRI could not be measured. Third, our study measurements were recorded by a single examiner; ideally 2-3 examiners are needed. Last, further studies with larger sample population are required to understand the morphology better.

## Conclusions

The anteroposterior diameter of the ACL tibial footprint in Indian population is comparable to the western population. The tibial insertion site diameter averaged 15.40 mm and the center of the tibial insertion was found to be at an average of 43.51% along the Staubli and Rauschning line. The mean roof angle was 36.29 degrees and the ACL-bluemensaat angle averaged 4.70 degrees. Understanding of the tibial footprint morphology and the orientation of the ligament to the roof of the intercondylar notch help in anatomical graft placement during reconstruction.
